# Are Sponges Good Natural Sentinels for Monitoring Fish Diversity in Antarctic Coastal Waters?

**DOI:** 10.1002/ece3.72684

**Published:** 2025-12-16

**Authors:** Carlos Angulo‐Preckler, Marta Turon, Oriol Sacristan‐Soriano, Kim Præbel, Conxita Avila, Owen Simon Wangensteen

**Affiliations:** ^1^ Norwegian College of Fishery Science UiT The Arctic University of Norway Tromsø Norway; ^2^ Marine Science Program, Biological and Environmental Science and Engineering Division (BESE) King Abdullah University of Science and Technology (KAUST) Thuwal Kingdom of Saudi Arabia; ^3^ MNCN (CSIC) Museo Nacional de Ciencias Naturales Madrid Spain; ^4^ Àrea de Qualitat/Quality Area, Institut Català de Recerca de L'aigua ICRA/Catalan Institute for Water Research ICRA, Emili Grahit Girona Spain; ^5^ Technological Center in Biodiversity, Ecology and Environmental and Food Technology, TEC‐NIO Network University of Vic–Central University of Catalonia Vic Spain; ^6^ Department of Evolutionary Biology, Ecology, Environmental Sciences, and Biodiversity Research Institute (IrBIO), Faculty of Biology University of Barcelona Barcelona Catalonia Spain

**Keywords:** 12S rRNA, eDNA, metabarcoding, natural samplers, notothenioids, south Shetland Island, western Antarctic peninsula

## Abstract

Monitoring biodiversity in Antarctic ecosystems poses significant challenges, particularly due to the harsh environment. Traditional methods, such as beach seines, are time‐consuming, resource‐intensive, and difficult to carry out in Antarctica. To address these limitations, eDNA‐based techniques have emerged as a valuable alternative. We employed eDNA metabarcoding from water samples and sponges to assess coastal fish communities along the Western Antarctic Peninsula, aiming to explore species diversity across a latitudinal gradient. Our analysis identified 14 Antarctic fish species and one marine mammal. Although previous research has validated the use of sponge‐derived DNA for fish biodiversity studies, our results showed that seawater samples provided a very similar or, in many cases, an even more comprehensive view of the fish community. Interestingly, while sponge species exhibited variability in their performance, no significant differences were observed among them. Both water and sponge samples revealed similar beta diversity patterns, successfully capturing community composition at each location. The coastal fish fauna in the studied areas is highly dominated by notothenioids, with the genera *Notothenia*, *Lindbergichthys*, and *Trematomus* being the most abundant. All the species detected in both water and sponge samples were endemic to Antarctica, widely distributed, and previously known to inhabit the region. These findings are especially important given the increasing human activities, including commercial fishing and krill harvesting, that are impacting the Antarctic marine ecosystem.

## Graphical abstract

This study demonstrates that eDNA metabarcoding using both water and sponge samples is an effective tool for monitoring fish biodiversity along the Western Antarctic Peninsula. The results revealed 14 endemic Antarctic fish species and one marine mammal, with water samples often offering a more comprehensive view than sponges. These insights are especially valuable in light of growing human pressures—such as fishing and krill harvesting—that threaten the stability of Antarctic marine ecosystems.

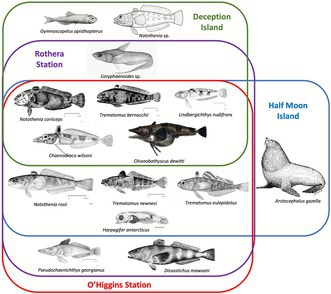

## 1 Introduction

The Southern Ocean (SO) covers about 10% of the Earth's oceans and has a rich geological history (Brooks et al. 2020). Interestingly, its fish diversity is relatively low compared to other continental shelf ecosystems, with only 322 documented species (De Broyer et al. 2014; Clarke and Johnston 1996). In contrast, Antarctic marine invertebrates exhibit notable richness and diversity (De Broyer et al. 2014; Clarke and Johnston 1996; Griffiths 2010). While cold temperatures are the major factor shaping the evolution of current fish species, other factors like isolation, habitat loss, and climatic cycles have also played significant roles (Clarke and Johnston 1996). The Antarctic fish fauna is largely defined by the diversification of a few groups, most notably the notothenioids. This group, which dominates the continental shelves, evolved from a single ancestor around 25 million years ago into more than 140 species, now accounting for approximately 90% of the fish biomass on Antarctica's continental shelf (Daane and Detrich 2022). The success of notothenioids has been attributed to their ability to exploit different prey types across various habitats due to evolved niche specialization (Targett 1981).

The Antarctic Circumpolar Current keeps the SO thermally isolated from northern waters (Barker and Thomas 2004). South of the Antarctic Polar Front, temperatures remain between −2 and +2°C throughout the year (Auger et al. 2021). The combination of low temperatures, strong currents, geographic isolation, and deep waters has resulted in high levels of species endemism in the SO (Eastman 2005). Of the 322 fish species found on the Antarctic continental shelf and upper slope, 222 species across 19 families are benthic (Eastman 2005). The most speciose taxa are Notothenioidei, Liparidae, and Zoarcidae, accounting for 88% of species diversity. Notably, the Notothenioidei alone account for 174 benthic or demersal species in the SO (Clarke and Johnston 1996). The relatively small number of species and limited diversity at higher taxonomic levels, compared to global fish diversity patterns, are defining characteristics of SO fish fauna (Eastman 2005). The Western Antarctic Peninsula (WAP) is a sea ice‐dependent ecosystem undergoing rapid environmental changes that are causing significant transitions (Turner et al. 2014; Ducklow et al. 2013). The impact of these changes on marine organisms and ecosystem processes remains poorly understood (Siegert et al. 2019), making it crucial to study fish species composition in the WAP's nearshore habitats for effective conservation and management. Based on seasonal sea‐ice oscillations, three ichthyofaunal zones have been identified in the SO, where sea ice is considered a key factor influencing the distribution and abundance of midwater fish communities (Kock 1992; Kellermann 1996).

Traditionally, many studies of fish communities in nearshore habitats have relied on beach seines or similar equipment (Ruhl et al. 2003; Grüss et al. 2021). However, these methods are time and resource consuming, requiring substantial infrastructure and personnel time (Steele et al. 2006), and are particularly challenging to implement in Antarctica. The extreme conditions of Antarctic waters make it difficult to gather quantitative data or conduct long‐term biodiversity monitoring using traditional approaches. Additionally, some traditional monitoring techniques have proven invasive to the species or ecosystems, such as marine surveys that involve highly destructive methods (Baldwin et al. 1996; Jones 1992). Alternatively, sampling and then analyzing environmental DNA (eDNA, (Thomsen et al. 2012)) from seawater or filter‐feeding organisms (natural samplers, (Mariani et al. 2019)) may produce similar datasets with substantially less effort (Jeunen et al. 2023; Neave et al. 2023).

Environmental DNA (eDNA) refers to the extracellular genetic material extracted from environmental samples (Bohmann et al. 2014). Its distribution in nearshore habitats is influenced by water movement and the rate of DNA degradation (Harrison et al. 2019). The portion of eDNA shed by organisms remains detectable in the environment for a limited time before it degrades due to chemical, physical, and biological factors (Holman et al. 2022). Although the decay rates of eDNA may vary between species, research suggests that eDNA generally remains detectable in seawater for up to 48 h (Holman et al. 2022; Collins et al. 2018). Biodiversity studies are increasingly using eDNA‐based methods to assess community patterns (Cordier et al. 2021; Dornelas et al. 2019). Metabarcoding of eDNA with taxon‐specific primers offers a way to overcome low detection probabilities, improving biodiversity estimates (Miya 2022; McElroy et al. 2020). These surveys consistently demonstrate the high sensitivity of eDNA techniques, enabling accurate species detection at a lower cost (Borrell et al. 2017; Deiner et al. 2017; Boussarie et al. 2018; Blackman et al. 2020). Traditional monitoring methods, on the other hand, face challenges such as difficulties in identifying cryptic species or juvenile stages, a decline in taxonomic expertise, non‐standardized sampling practices, and the invasiveness of some survey techniques. Thus, eDNA presents an efficient, non‐invasive, and easy‐to‐standardize alternative (Thomsen and Willerslev 2015). When paired with advanced and cost‐effective DNA sequencing technologies, eDNA holds significant potential for addressing the challenges of biomonitoring in Antarctic ecosystems. In many cases, fish species remain undetected due to ineffective direct capture or visual surveys, particularly in the shallow waters of Antarctica. High‐throughput sequencing (HTS) of macro‐organismal DNA from environmental samples provides a cutting‐edge conservation method to assess ecological communities (Deiner et al. 2017; Thomsen and Willerslev 2015). This approach enhances traditional biodiversity monitoring by targeting eDNA, which is typically more widespread than the organisms themselves, allowing for the detection of rare and elusive species (Bakker et al. 2019; Jerde et al. 2019).

Filter‐feeding organisms such as sponges have been recently proposed as ideal ‘natural samplers’ to retrieve eDNA for aquatic biodiversity monitoring (Mariani et al. 2019; Turon et al. 2020). This is due to their ability to recover and accumulate extra‐organismal eDNA through their filtering activity, as sponges are among the most efficient water‐filtering organisms on Earth (van Soest et al. 2012). Therefore, sampling sponge fragments and extracting their natural‐sampler DNA (nsDNA) is a promising approach that could potentially become more efficient than mechanical filtering of seawater. However, further experimental tests are still needed to validate the suitability of nsDNA extracted from sponges to accurately recover the vertebrate biodiversity patterns in different environmental conditions across the Globe and to compare the results from sponges' nsDNA with those from eDNA filtered from seawater from the same locations.

In the present study, we aim to (1) validate the suitability of natural‐sampler DNA (nsDNA) extracted from Antarctic sponges to recover fine‐scale fish diversity in coastal water from the WAP, and (2) compare the fish inventories resulting from nsDNA of different sponge species to those obtained from two pore‐size fractions of filtered seawater (eDNA) from the same locations, with the overall aim of working towards the validation of eDNA and nsDNA studies for fish biodiversity monitoring in Antarctic ecosystems.

## 2 Methods

### 2.1 Sample Collection and Study Site

**FIGURE 1:**
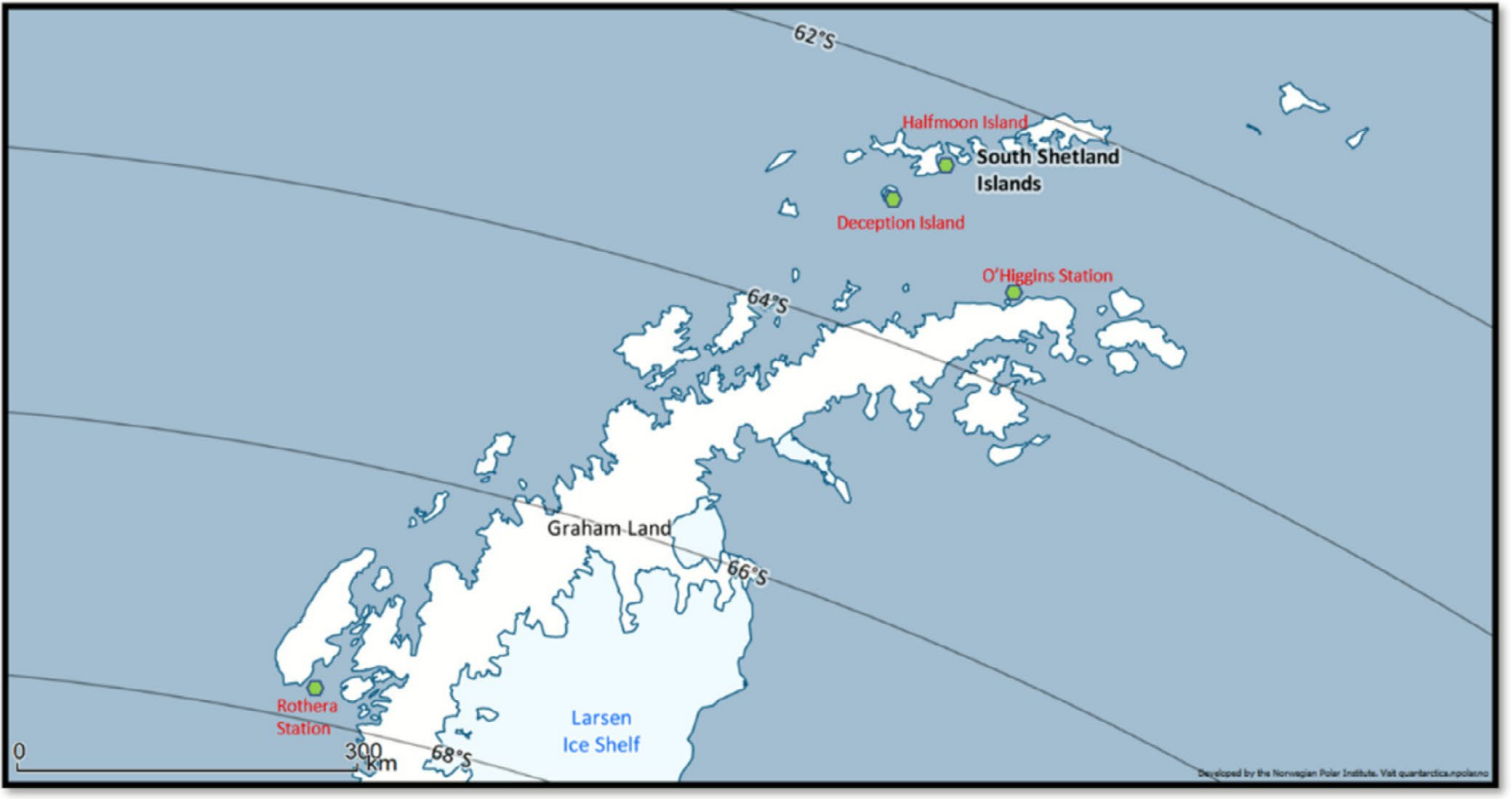
Map of the sampling locations. Map constructed with QGIS software (v. 3.16) with Quantarctica package.

Four different sponges: *Dendrilla antarctica* Topsent, 1905, *Sphaerotylus antarcticus* Kirkpatrick, 1907, *Mycale (Oxymycale) acerata* Kirkpatrick, 1907, and *Hemigellius pilosus* (Kirkpatrick, 1907) were selected based on their local abundance and representativeness within the study sites. All sponges were collected by SCUBA diving at depths ranging from 15 to 20 m across the four sampling locations during the austral summer of 2016. To minimize cross‐sample contamination during collection, each sponge specimen was immediately placed into an individual sterile ziplock bag underwater before being brought to the surface. Two stations from the South Shetland Islands (Deception and Halfmoon islands) and two stations from the Western Antarctic Peninsula (WAP), near the vicinities of O'Higgins and Rothera research stations (Figure 1). Three replicate seawater samples (2 L each) were collected next to the sampled sponges from all locations using sterile containers. Seawater samples were sequentially passed through polycarbonate 5 and 0.22 μm filters (MilliporeSigma, Burlington, MA, United States) to avoid clogging the ones with the smaller pore diameter. Filtration was performed shortly after collection under a laminar flow hood sterilized with UV light prior to each filtration session to minimize the risk of contamination. All filters and sponge tissue samples were preserved in RNAlater until DNA extraction. Available DNA extracts from sponge tissues and seawater filters were also used previously for research on the microbiome structure (Sacristán‐Soriano et al. 2020) and were included in the present study as opportunistic samples for the analysis of the Antarctic fish community and to evaluate the efficiency of sponges as natural samplers.

### 2.2 DNA Extraction, PCR Amplification and Sample Sequencing

DNA extractions from small fractions (0.5 cm^3^) of sponge samples or polycarbonate filters were performed using the DNeasy Blood & Tissue Kit (Qiagen). In total, 61 samples, 25 water samples, and 36 sponge samples (Table 1) were used in this study (61 samples plus a blank with 3 PCR replicates each). Total DNA extracted was amplified using the high‐specificity MiFish primer set targeting a hypervariable region in the mitochondrial 12S rRNA gene (163–185 bp), which amplifies vertebrates, and specifically fishes (Miya et al. 2015). Three PCR replicates were run per sample as technical replicates, with two negative controls without DNA template. The thermocycler profile consisted of 94°C for 10 min; 40 cycles × (95°C/30 s, 60°C/30 s, 72°C/30 s); 72°C/5 min. To tag individual samples, a multiplex 7‐bp identifier tag was attached to forward (5′‐GTCGGTAAAACTCGTGCCAGC‐3′) and reverse (5′‐CATAGTGGGGTATCTAATCCCAGTTTG‐3′) primers. The success of PCR amplifications was checked by gel electrophoresis in 1% agarose, and PCR products were pooled together into three multiplex sample pools. Equal volumes of each tagged PCR product (10 μL) were pooled and purified using MinElute PCR purification columns (Qiagen), where fragments below 70 bp were removed. Library preparation was performed with the NEXTflex PCR‐free library preparation kit (BIOO Scientific). Exact library concentrations were measured by qPCR with the NEBNext Library Quant Kit (New England BioLabs). All genetic lab work was performed following clean lab routines at the Norwegian College of Fishery Science. Sequencing was then performed using a partial run of an Illumina NovaSeq (2 × 150 bp) by Novogene Europe (Cambridge, UK).

**TABLE 1:** Data of the sampling locations, sample type, sponge species, biological and technical replicates, and detection rate (percentage of PCR replicates with enough reads to be included in the analysis).

Locality	Latitude	Longitude	Sample type	Species	Biological replicates	Technical replicates	Detection rate
Deception Island	−62.984002	−60.562240	Sponges	*Dendrilla antarctica*	3	9	77.8
				*Sphaerotylus antarcticus*	4	12	8.3
				*Hemigellus pilosus*	4	12	41.7
				*Mycale acerata*	4	12	41.7
			Water	Filter 5 μm	3	9	22.2
				Filter 0.2 μm	3	9	22.2
Half Moon Island	−62.593079	−59.906964	Sponges	*Dendrilla antarctica*	4	12	66.7
				*Sphaerotylus antarcticus*	3	9	88.9
			Water	Filter 5 μm	3	9	55.6
				Filter 0.2 μm	4	12	25.0
O'Higgins Research Station	−63.320612	−57.905138	Sponges	*Dendrilla antarctica*	4	12	50.0
				*Sphaerotylus antarcticus*	2	6	50.0
			Water	Filter 5 μm	3	9	55.6
				Filter 0.2 μm	3	9	44.4
Rothera Research Station	−67.565397	−68.118247	Sponges	*Dendrilla antarctica*	4	12	58.3
				*Sphaerotylus antarcticus*	4	12	41.7
			Water	Filter 5 μm	3	9	44.4
				Filter 0.2 μm	3	9	33.3

### 2.3 Bioinformatics

The MJOLNIR pipeline (https://github.com/uit‐metabarcoding/MJOLNIR) was used for the bioinformatic analyses. Paired‐end reads were aligned using *illuminapairedend* and only sequences with alignment quality score > 40 were kept. Demultiplexing was done with *ngsfilter*, which also removed primer sequences. Aligned reads with a length of 140–190 bp and without ambiguous positions were selected using *obigrep* and then dereplicated with *obiuniq*. Chimeric sequences were removed using the uchime_denovo algorithm implemented in vsearch v1.10.1 (Rognes et al. 2016). Clustering of sequences into molecular operational taxonomic units (MOTUs) was performed using the SWARM 2.0 algorithm (Mahé et al. 2015) with a d value of 2. Taxonomic assignment of the most abundant (representative) sequence of each MOTU was done with the *ecotag* algorithm using the default parameters (Boyer et al. 2016) against the DUFA_MiFish v2020‐05‐01 reference database (https://github.com/uit‐metabarcoding/DUFA), compiled from sequences of the MiFish fragment available from GenBank. Further refining of the dataset consisted of removing MOTUs identified as human or terrestrial vertebrates, probably arising from contaminations, and applying a minimal abundance threshold per MOTU of 10 reads.

### 2.4 Community‐Level Analysis

Statistical analyses were performed in R version 4.0.5 with the *vegan* package (Oksanen et al. 2019) and graphic visualization was done with the *ggplot2* package (Wickham et al. 2016). Biodiversity indices (MOTU richness, Shannon‐Weaver index, Simpson index, and total reads) were calculated. Analyses of variance (ANOVA) were used to detect differences in diversity metrics among sample type, sponge species, and localities. Data were converted to relative read abundance to build a Bray–Curtis dissimilarity matrix, which was used to assess the variance in community composition using Permutational Multivariate Analyses of Variance (PERMANOVA). All PERMANOVAs were performed after square root transformation of relative read abundance data and were tested using the *adonis* function with 1000 permutations. Significant differences among sponge and water samples were assessed with a one‐way PERMANOVA, with the factor *sample type* (sponge and filter). Differences between sponge species and location were assessed using a two‐way PERMANOVA, with factors *species* ( *D. antarctica* , *S. antarcticus* , *M. acerata*, and *H. pilosus* ), *location* (Deception Island, Half Moon Island, O'Higgins, and Rothera), and an interaction term. Pairwise comparisons were subsequently conducted for all significant PERMANOVA results. Additionally, PERMDISP analysis (*betadisper* function) was used to detect differences in homogeneity (dispersion) among groups for all significant PERMANOVA outcomes. Non‐metric multidimensional scaling (nMDS) representation with Bray‐Curtis dissimilarities was performed with the *metaMDS* function with 500 iterations. Three different nMDS were plotted to visualize differences in community composition among *sample type* and within *locations* for water and sponge samples. A heatmap was used to represent species with 0, 1, 2, and 3 detections per sample. To compare fish community profiles, a Venn diagram from presence/absence MOTU data was constructed. A bubble chart was also constructed from MOTU relative abundance to plot community dissimilarities among locations. Finally, although both filters from the same water sample (5 μm and 0.22 μm) were merged for the community analyses, they were also analyzed separately to compare their efficiency in detecting MOTUs. To assess the consistency of fish DNA detection, the detection rate for each sample was calculated as the number of PCR replicates with a positive fish DNA signal divided by the total number of technical replicates. Pearson's Chi‐squared tests were subsequently applied to evaluate whether detection rates differed significantly among sample types (Porifera vs. water), sponge species, and collection localities. Additionally, to assess the consistency of detections among biological and technical replicates, we calculated the coefficient of variation (CV) for each sample type (Porifera and water) across localities. The CV was computed as the ratio of the standard deviation to the mean of read abundance among replicates, providing a measure of relative dispersion. Statistical differences in both total read counts and CV values between sample types and among localities were evaluated using the Wilcoxon rank‐sum test. These metrics allowed us to evaluate both the variability and reproducibility of molecular detections across sample types and sites.

## 3 Results

### 3.1 Community Richness

**FIGURE 2:**
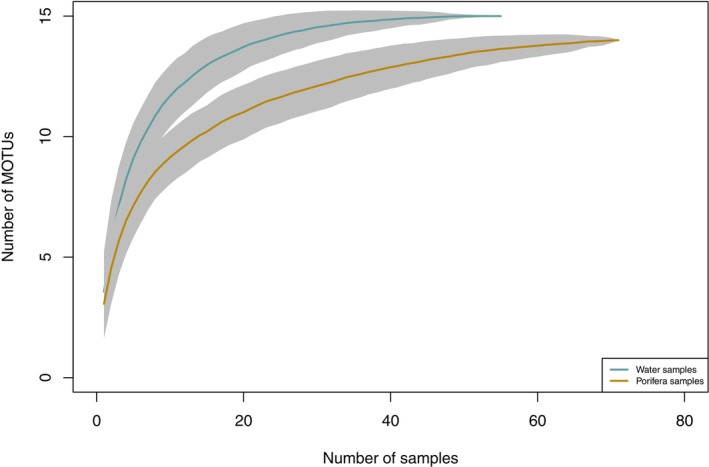
Species accumulation curves with 95% confidence intervals (gray areas), illustrating the accumulation of species diversity for water and sponge samples separately. The curves were generated from data obtained from all 183 samples included in the study, accounting for three PCR replicates per sample.

A total of 10,074,592 reads were obtained after demultiplexing and quality filtering (4,480,295 and 5,594,297 reads from sponge and water samples, respectively), with a mean sequencing depth of 90,868 ± 156,456 reads per sample (Table S1). Our final dataset consisted of 15 molecular operational taxonomic units (MOTUs) assigned to vertebrates, recovered from 183 PCRs considering both sponge and water samples (Table 1). Of these, 71 out of 108 sponges and 55 out of 75 water samples had positive detections of at least one MOTU (in order to be conservative and avoid false positives, samples with less than 10 reads after the removal of contaminants were removed from the analysis). Although there is a high variability in the number of reads per sample, the species accumulation curves showed that sampling depth was enough to detect the MOTU richness as a proxy for species richness (Figure 2). The sequencing depth also showed a high correlation (*R* ^2^ = 0.93) between the total number of detected MOTUs and the number of total reads, with a greater efficiency of the water samples compared to the sponge samples (Figure S1). However, the high difference in the number of reads between replicates suggests an important stochastic component of the PCR amplifications. Each sample recovered several MOTUs (S = 3.9 ± 1.9), despite the low number of total MOTUs detected in the entire dataset. The maximum MOTU richness was 12 in a sample (joining the replicates) and 10 different MOTUs in a single replicate (Figure S2).

**FIGURE 3:**
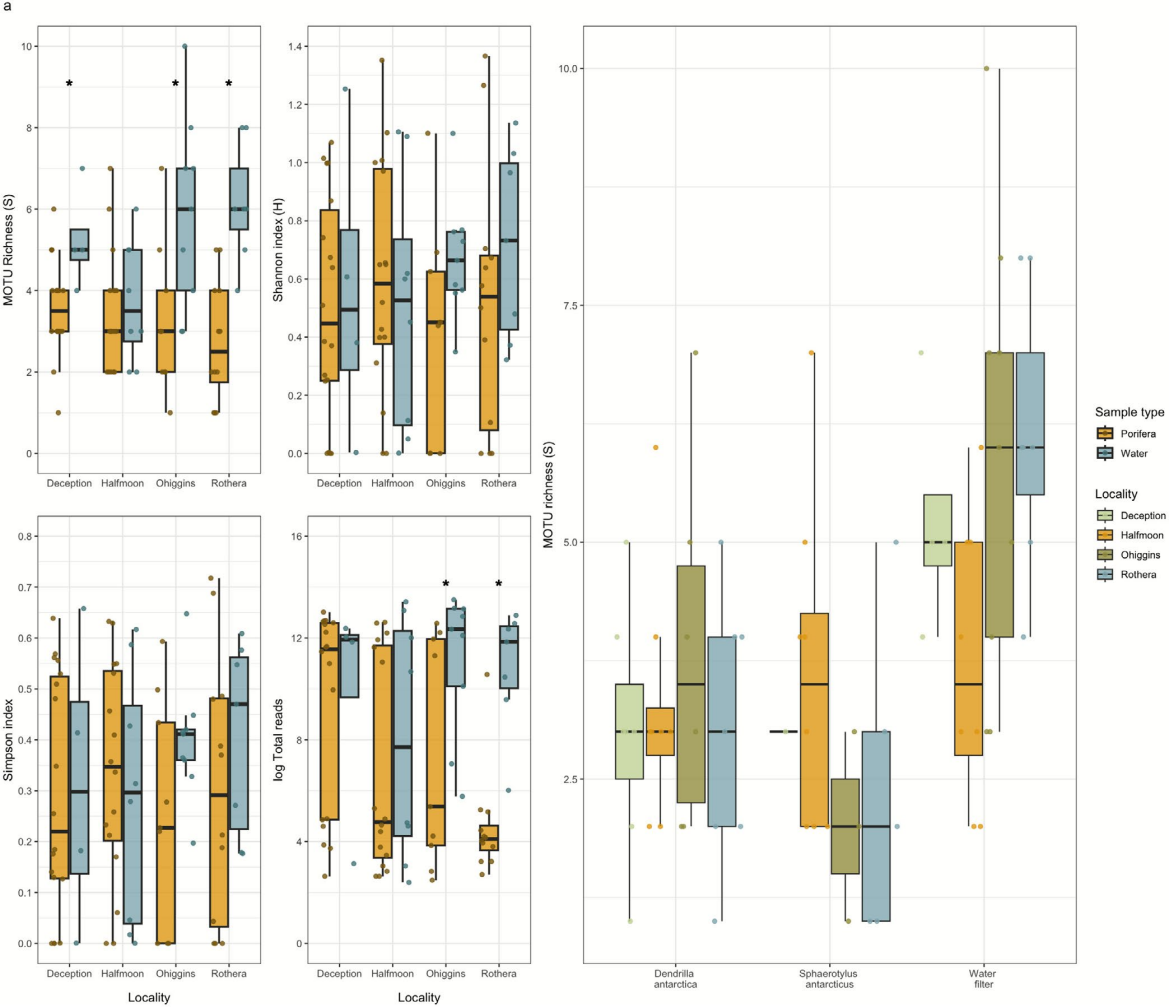
Biodiversity indices (a) MOTU Richness, Shannon index, Simpson index, and Total reads by locality and sample type, and (b) MOTU richness by sample type.

Significant differences were observed in the MOTU richness recovered from water and sponge samples (*F*_1,81_ = 23.97, *p* < 0.001), with the former exhibiting higher values than the latter. Despite significant differences being found in MOTUs richness in three out of four localities, the high variability between samples resulted in no differences between the remaining diversity indices measured (Figure 3, Table S2). On the other hand, both pore size fractions also showed significant differences but only in species richness (*F*_1,53_ = 4.905, *p* = 0.0311). Thus, the 5 μm filter recovered a mean of 4 ± 1.73 MOTUs per sample, while the 0.22 μm filter recovered 3.04 ± 1.73 MOTUs per sample (Figure S3).

We compared the total number of reads between Porifera and water samples across localities. Water samples generally displayed higher read counts in two of the four localities (O'Higgins and Rothera; *p* < 0.05), whereas in the remaining sites, read abundances were similar or slightly higher in Porifera (Figure S4). Furthermore, the coefficient of variation (CV) across biological and technical replicates indicated moderate variability. Most technical replicates exhibited consistent read counts, while biological replicates showed greater variation, indicating that observed differences primarily reflect natural variation rather than technical artifacts. Consistent with the Wilcoxon test results, no significant differences were detected, although water samples tended to exhibit slightly higher dispersion compared to Porifera (Figure S5), suggesting greater variability in eDNA concentrations among replicates. Detection rates across samples ranged from 8% to 89%, with an average of approximately 55%, indicating overall reproducible amplification success across technical replicates (Table 1). Detection rates did not differ significantly across sample types (Porifera vs. water; *χ* ^2^ = 18, df = 11, *p* = 0.082), sponge species (*χ* ^2^ = 49.5, df = 55, *p* = 0.684), or localities (*χ* ^2^ = 40, df = 33, *p* = 0.187), indicating no consistent spatial or sample‐type patterns in fish DNA detection. These results indicate that sequencing depth and amplification success were generally comparable between both sample types, supporting the reliability of the metabarcoding data used for subsequent biodiversity analyses.

### 3.2 Community Structure

**FIGURE 4:**
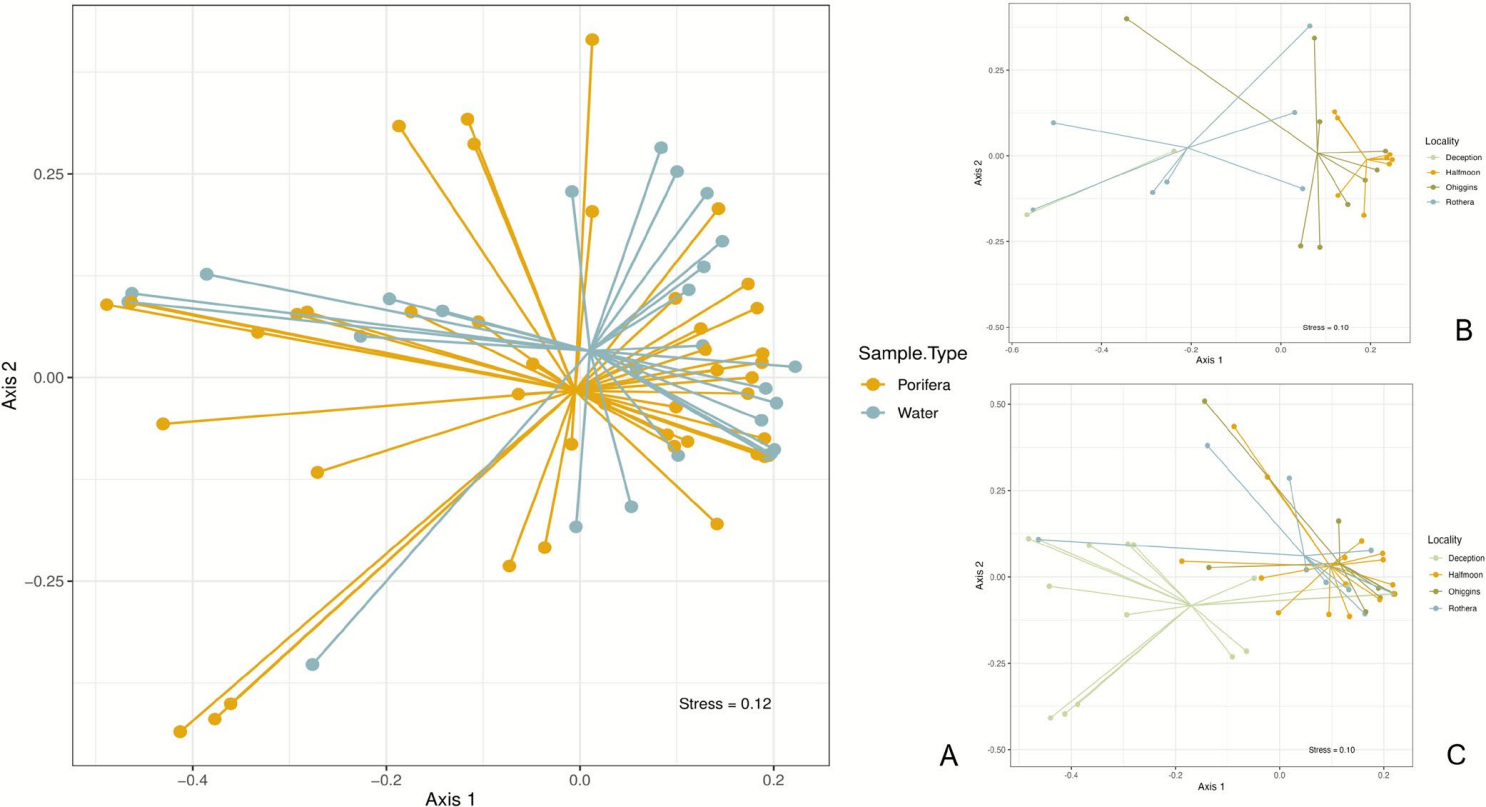
Non‐metric multidimensional scaling (nMDS) ordination of fish communities obtained based on Bray‐Curtis distances. Colors show differences between (a) sample type, and (b) localities for water samples, and (c) localities for sponge samples.

The non‐metric multidimensional scaling (nMDS) representation showed an important overlap between the fish community recovered from sponges and water samples (Figure 4a). All fish species detected in the water samples were also recovered in the sponge samples. The only MOTUs not detected in the sponge samples were *Arctocephalus gazelle*, the Antarctic fur seal. PERMANOVA analysis of the factor *sample type* (*water* vs. *sponges*) showed significant differences (a*donis*: *F*_1,109_ = 1.87, *p* = 0.027), while no differences were detected among factor *species* (*sponges*) (*adonis*: *F*_4,106_ = 1.33, *p* = 0.058).

Slight differences were observed in the community composition along the WAP (Figure 4b,c), exhibiting statistically significant differences in the community structure recovered from both water and sponge samples. Specifically, when analyzing sponge samples, only Deception Island showed a clear distinct community structure. However, with water samples, the differences appear to be more pronounced, suggesting a potentially broader impact across multiple localities. The 2‐way PERMANOVA with *species* and *locality* as factors showed significant differences, but these factors only explained a low amount of the total variation (Table S3). The PERMDISP test supported a homogeneous group dispersion displaying not significant values. Furthermore, the pairwise comparison focused on the differences between water samples and the sponge *Sphaerotylus antarcticus* (*p* = 0.002, *R* ^2^ = 0.077) concerning the factor *species*, and between Rothera and O'Higgins stations (*p* = 0.012, *R* ^2^ = 0.066) concerning the *locality* factor.

### 3.3 Antarctic Fauna

**FIGURE 5:**
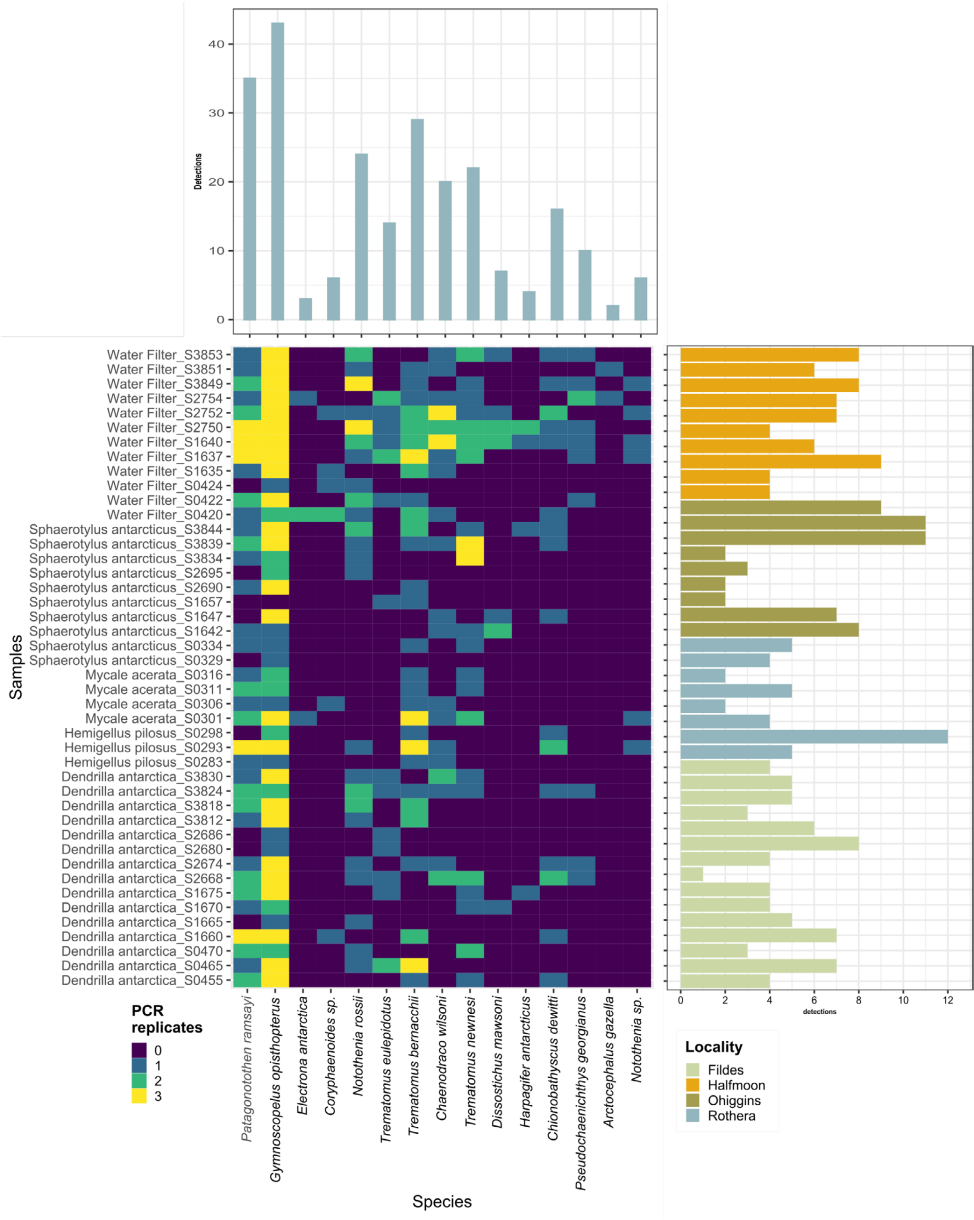
Heatmap based on presence‐absence data. Scale color indicates whether a MOTU is detected in 0, 1, 2, or 3 replicates of a sample. Sponge and water samples (y‐axis), and fish species (x‐axis), are ordered according to cluster dendrogram based on Jaccard distances. Bars indicate total detections of fish species in all samples (upper panel), and fish species in each sample (right panel). Bar color for y‐axis indicates the sampling locations.

**FIGURE 6:**
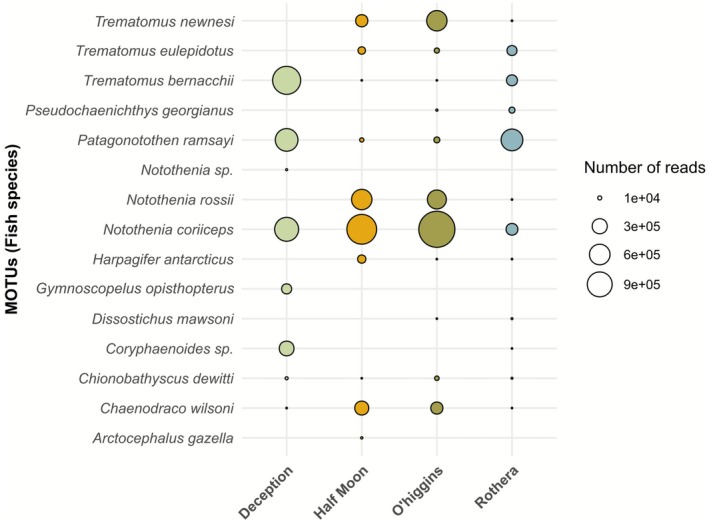
Bubble plot showing Antarctic fish and mammal species detected among localities, where the size of the bubble indicates the MOTU relative abundance of read counts.

Most of the MOTUs were identified at the species level (13 MOTUs), with only two MOTUs identified at the genus level. As each MOTU belonged to a different species, both terms will be used interchangeably hereafter. For the whole dataset, we identified 14 different Antarctic fishes and one marine mammal (the Antarctic fur seal), grouping six different families: Nototheniidae (8 species), Channichthyidae (3 species), and Macrouridae, Myctophidae, Harpagiferidae, and Otariidae, with only one species each. Thus, the Nototheniidae family represented more than 90% of the total reads recovered (Figure S6), followed by the Channichthyidae family (5% of the total reads). The majority of MOTUs were detected in several samples and locations. *Notothenia coriiceps* and *Lindbergichthys nudifrons* (previously referred to as *Lepidonotothen nudifrons* ) were the most frequent species occurring across samples (with 54 and 41 detections, respectively), and also had the highest number of reads (Figure 5). Due to the high variability in the number of reads recovered by sample, after they were pooled by locality, only MOTUs with 10 or more reads were considered as positive detections (Figure S7). A fish core community shared among all localities was detected, composed of five different species ( *Notothenia coriiceps* , *Lindbergichthys nudifrons*, *Trematomus bernacchii* , *Chaenodraco wilsoni* , and *Chionobathyscus dewitti* ). Conversely, three species were exclusive to just one location (the fish *Notothenia* sp. and *Gymnoscopelus opisthopterus* at Deception Island, and the fur seal *Arctocephalus gazelle* at Half Moon Island). Half Moon Island and O'Higgins station shared the most similar fish communities, both in diversity (species richness) and in ‘*abundance*’ (total reads). They shared similar ‘*abundance*’ values of *Notothenia coriiceps*, *N. rossii*, *C. wilsoni*, and *Trematomus newnesi*. Deception Island differentiated itself from the other locations by the important contribution of *T. bernacchii* and the presence of *Coryphaenoides* sp. and *Gymnoscopelus opisthopterus* . Finally, Rothera station presented the richest fish community but with the lowest ‘*abundance*’ values (Figure 6). Overall, the majority of fish detected in the samples were demersal species, related to shallow habitats with some remarkable exceptions such as the pelagic *Dissostichus mawsoni* and the bathydemersal *Chionobathyscus dewitti* and *Chaenodraco wilsoni* .

## 4 Discussion

### 4.1 Water Versus Sponge Samples

The rapid development of high‐throughput sequencing technologies has allowed the use of molecular biodiversity assessment as a viable alternative to morpho‐taxonomy inventories in aquatic ecosystems (Bohmann et al. 2014; Pawlowski et al. 2022). Most metabarcoding studies have focused on water eDNA. However, eDNA metabarcoding has also been applied to bulk and sediment samples to assess the ecological status of aquatic ecosystems (Antich et al. 2021; Hestetun et al. 2021). Recently, sponges have been proposed as ideal candidates to act as natural environmental samplers (nsDNA) (Mariani et al. 2019; Turon et al. 2020). In this study, we used specific primers for vertebrates (Miya et al. 2015) to unveil fish diversity from nsDNA retrieved from tissues of Antarctic sponges and compared them to that obtained from filtered sea water samples. We sampled at four localities in the WAP, covering a spatial scale over 500 km. We were able to identify up to 14 fish and one seal species. Despite the low richness values, species accumulation curves showed that the sampling effort adequately captured the fish diversity within the studied area. Both curves demonstrate that the sampling effort was enough, with slightly better results in the water samples curve. An important proportion of our samples (31%) did not retrieve any target PCR detection, or the counts contained too few reads to be included in the analysis. This could be attributed to methodological issues during library construction and sequencing, such as deviations from equimolar concentrations among samples or replicates related to our pooling and multiplexing strategy, which similarly affected both sponge and seawater samples. Alternatively, we cannot exclude the possibility that fish DNA was genuinely absent in some replicates. Notably, the failed replicates were not consistently associated with particular locations, supporting the interpretation that at least part of the missing data stemmed from the stochastic nature of trace‐DNA. Although fish nsDNA was not ubiquitous across all samples, we observed a comparable rate of positive detections from sponges and filtered water samples (65.7% and 73.3%, respectively), consistent with previous observations (Mariani et al. 2019; Turon et al. 2020). This means that stochasticity seems to be inherent to the methodology but is not affected by sample type. This limitation could probably be overcome by increasing the sample volume of the sponge tissue to increase the probability of detecting nsDNA (Turon et al. 2020). Differences in the efficiency of capturing fish environmental DNA among sponge species were not observed. Although sponges contained low values of MOTU richness, we did not find any sponge species to be a “better” natural sampler than the others (Table S2). While sample sizes were uneven among sponge species (Table 1), all specimens were collected during a single dive at Deception Island under comparable environmental conditions, allowing a controlled comparison of their performance as natural samplers. The proportion of samples yielding positive detections varied notably among species: *D. antarctica* (7/9 technical replicates), *S. antarcticus* (1/12), *H. pilosus* (5/12), and *M. acerata* (5/12), suggesting species‐specific differences in DNA capture efficiency, although statistical comparisons did not detect significant differences in the fish communities retrieved (Table S2). Water movements can highly affect the distribution of eDNA in the environment (Goldberg et al. 2016), but we were able to discriminate community composition between filtered water and sponge samples, discarding that the lack of specific patterns between sponge species was due to fine‐scale sampling (both water and sponge samples were collected in the same dive at each location). The only difference found in the retrieved community composition was between the sponge *Sphaerotylus antarcticus* and the water samples. While comparing sponge species, the differences were not pronounced enough to be detected. However, upon including the water samples, some differences emerged. It seems reasonable that the differences have occurred between the most efficient samples (filtered water) and the only sponge with a different morphology and lower species richness values. This dome‐shaped sponge with several elongated papillae present on the surface was the only non‐massive sponge species, probably conditioning its filtering features (Plotkin et al. 2017).

The ability of the DNA shed from macroscopic organisms to persist in the environment, where it can be sampled, extracted, and analyzed, has entailed a major technological and scientific breakthrough within the last decade. Although previous research validated the use of DNA recovered from sponges to discern fish biodiversity patterns (Turon et al. 2020; Jeunen et al. 2024), results comparing the effectiveness of sponges versus seawater samples are mixed. Some research suggests similar detection rates between the two methods (Jeunen et al. 2023; Cai et al. 2022), while others report better performance of sponges for certain fish groups (Cai et al. 2024). Several studies, including (Mariani et al. 2019; Jeunen et al. 2024), have focused exclusively on natural or sponge samples without parallel water sampling. While Mariani et al. (2019) were pioneering in demonstrating the potential of such samples for detecting Antarctic vertebrates through eDNA analysis, differences in sampling design—particularly the absence of water samples and replication—limit the extent to which their findings can be directly compared with our systematic and replicated water‐based eDNA survey. Our study found that sponges were less effective than sea water samples in recovering fish community richness in most of the studied locations. Therefore, while sponges can serve as indicators, they do not enhance the results obtained from water samples. This pattern may be influenced by the low fish species richness typical of Antarctic shallow waters, which can reduce detectable differences between sampling methods. Additionally, considering that sampling sponges is an invasive method of collection, seawater samples are recommended. This contrasts with the situation in benthic communities, where sea water samples appear to be less effective than bulk samples (Antich et al. 2021). Despite variations in sponge species' performance noted in previous studies (Neave et al. 2023; Cai et al. 2022), no significant differences were observed among different sponge species in our study. The observed slight differences may be attributed to variations in sponge physiology, suggesting a direct correlation with the efficiency of nsDNA (Neave et al. 2023). The fact that 2 L of water contribute better to discriminate fish communities through metabarcoding than several sponge samples grouped together, each one with the ability to filter up to 10,000 L of water per day (Kahn et al. 2015), seems to be related to something else rather than just the persistence of DNA in seawater (Holman et al. 2022). Temporal factors had significant effects on eDNA concentration, whereas fish species and feeding factors did not (Murakami et al. 2022). The accuracy of taxonomic and functional profiles of microbial communities has been validated using just 1 μL of seawater (Bramucci et al. 2021). Consequently, our findings indicate that filtering large volumes of seawater might not be necessary for the retrieval of eDNA from marine environments. Although with only a few sponge species we were able to obtain a good coverage of the fish community, filtered water samples have been probed to enhance this coverage. Thus, probably with less than 2 L of filtered sea water, consistent results could be achieved. Moreover, sea water samples are easier to collect and do not involve any disturbance of the natural environment, making them the perfect samples for biomonitoring fish communities in cold water environments. On the other hand, sequential filtering showed that eDNA exists in a range of particle sizes, throwing very similar values when testing the efficiency of the two pore sizes we tested. These results were consistent with previous metabarcoding studies where similar results were obtained from different pore size filters (Li et al. 2018; Deiner et al. 2018). There is a trade‐off between filtration time needed to attain a specific volume and the pore size interacting with the particulate matter in water samples clogging pores. Thus, as larger pore sizes allow for shorter filtration times, our results suggest more efficiency with the 5 μm filter.

Some studies showed that eDNA quantity (number of reads) could be a proxy for the abundance or biomass of macro‐organisms (Guri et al. 2023; Ushio et al. 2018; Yamamoto et al. 2016). Nonetheless, the general use of eDNA to estimate fish abundance/biomass is still controversial because factors that influence eDNA quantity, such as eDNA decay rates in seawater and their release rates, are likely to depend on the ecology and physiology of target species and other biotic/abiotic factors (Maruyama et al. 2014; Tsuji et al. 2017). Nevertheless, some evidence suggested that the quantity of eDNA may be used as a “rough index” for abundance/biomass (Ushio et al. 2018), although careful interpretations are necessary.

### 4.2 Fish Composition in the Western Antarctic Peninsula

To the best of our knowledge, SCUBA‐diver‐based underwater visual censuses, the most widely used method to collect data on coastal fish assemblages (Brock 1954), have never been performed in subtidal Antarctic waters. Due to logistic constraints, low fish densities, and low detectability, very scarce information is available for nearshore Antarctic waters. Vessels are unable to trawl at depths shallower than 50 m due to the close vicinity to land and most of the Antarctic inshore waters are still unmapped. In recent years, the development of novel video‐based tools for assessing fish assemblages has emerged for an array of habitats (Mallet and Pelletier 2014), but only a few underwater vehicles have been used in Antarctica so far (Cazenave et al. 2011; Smith et al. 2012), and never to study fish communities. Due to the fact that our ability to observe macroscopic organisms living in aquatic ecosystems is limited, eDNA metabarcoding offers a reliable sampling method with a high probability of detection. Fish eDNA approaches can be a powerful tool for non‐invasive monitoring of nearshore Antarctic habitats. The coastal fish fauna in the studied places is highly dominated by notothenioids, with the genera *Notothenia*, *Lindbergichthys*, and *Trematomus* being the most abundant. In line with most of the Antarctic studies, family Nototheniidae dominate both the diversity and biomass of the Antarctic fish communities along the WAP nearshore waters (Targett 1981; Friedlander et al. 2020). Although the collected samples had an inshore distribution, some of the fish species retrieved here have deeper preferences ( *Chionobathyscus dewitti* , *Chaenodraco wilsoni* , and *Trematomus eulepidotus* ), and some of them are very elusive to detect during diving operations (author's personal observations). Most of the species recovered are demersal or benthopelagic species (Table 2). However, we also detected some purely pelagic species, such as *Dissostichus mawsoni* , the Antarctic toothfish, present in both stations of the WAP (O'Higgins and Rothera). Catches of *Dissostichus mawsoni* are scarce around the continent, and records frequently come from the stomachs of seals or sperm whales (Yukhov 1971; Eastman and Barry 2002). The occurrence of pelagic fish in coastal waters is rarely documented and could be attributed to the temporal introduction of nekton from adjacent offshore areas (Barrera‐Oro 2002). It is known from other regions that juvenile fishes are abundant in shallow nearshore waters before they undergo settlement to the demersal phase of life (Everson 1969), reinforcing the importance of nearshore waters as nurseries. Inshore‐based ichthyological studies in the WAP (including the Scotia Arc) deal almost exclusively with demersal notothenioid fish. Some studies describing the ichthyofauna from inshore waters only found a few species (commonly < 6) (Kock 1992; Skora 1993), with an increase in species richness with depth, a characteristic tendency in Antarctic waters (Kock and Stransky 2000). It is noteworthy that, with a spatial and temporally restricted sampling of 15 sponge and 3 sea water samples at Deception Island (one single dive), we have detected similar values of fish diversity to those found in other studies along five different cruises (Ruhl et al. 2003). This underscores the greater efficiency of eDNA studies compared to traditional methods, as they achieve comparable results with significantly less sampling effort. Despite having carried out multiple opening/closing net and environmental system (MOCNESS), otter trawls, bottom camera sled‐based line‐transect photography, and remotely operated vehicle (ROV) video, they only found ten different fish species. If data were standardized by their cost‐efficiency, the advantage of metabarcoding is overwhelmingly clear. Poor species overlap between our data and these studies is likely due to different sampling depths, with only two species detected in both studies ( *T. bernacchii* and *L. nudifrons* ). We also detected the genetic signal of *Parachnenichthys charcoti* at Deception Island, but although this species was removed after the data cleaning process, we know that it is present in shallow waters of Deception Island (author's personal observation).

**TABLE 2:** Taxonomic information for the MOTUs recovered in this study. The table includes the identity supporting each taxonomic assignment (Identity), the number of samples in which each MOTU was detected (Detection), the total number of reads assigned to each MOTU (Reads), and the depth range of each species obtained from FishBase.

Family_name	Scientific_name	Identity	Detections	Reads	Depth range	Habitat	Common name
Harpagiferidae	*Harpagifer antarcticus*	1	4	74,671	0–5	Demersal	Antarctic spiny plunderfish
Channichthyidae	*Chaenodraco wilsoni*	1	20	437,586	200–800	Bentho‐pelagic	Spiny icefish
Channichthyidae	*Chionobathyscus dewitti*	0.9941	16	18,919	500–2000	Bathy‐demersal	—
Channichthyidae	*Pseudochaenichthys georgianus*	1	10	34,259	0–475	Demersal	South Georgia icefish
Macrouridae	*Coryphaenoides* sp.	1	6	286,646	—	—	—
Myctophidae	*Gymnoscopelus opisthopterus*	1	3	119,634	550–900	Bathy‐pelagic	—
Nototheniidae	*Lindbergichthys nudifrons*	1	41	1,457,011	3–400	Demersal	Yellowfin notie
Nototheniidae	*Notothenia coriiceps*	1	54	4,241,879	0–500	Demersal	Black rockcod
Nototheniidae	*Notothenia rossii*	1	24	1,091,419	5–350	Demersal	Marbled rockcod
Nototheniidae	*Trematomus eulepidotus*	0.9415	14	197,033	70–650	Demersal	Blunt scalyhead
Nototheniidae	*Trematomus bernacchii*	1	29	1,304,288	0–700	Demersal	Emerald rockcod
Nototheniidae	*Trematomus newnesi*	1	22	772,868	0–400	Demersal	Dusky rockcod
Nototheniidae	*Dissostichus mawsoni*	1	7	454	0–2200	Pelagic	Antarctic toothfish
Nototheniidae	*Notothenia* sp.	0.9474	7	631	—	Demersal	—
Otariidae	*Arctocephalus gazella*	1	2	1010	0–300	—	Antarctic fur seal

*Note:* Detection: number of samples where MOTUs were present. Reads: total reads per MOTUs. Depth range: (m) data from FishBase (www.fishbase.se).

**FIGURE 7:**
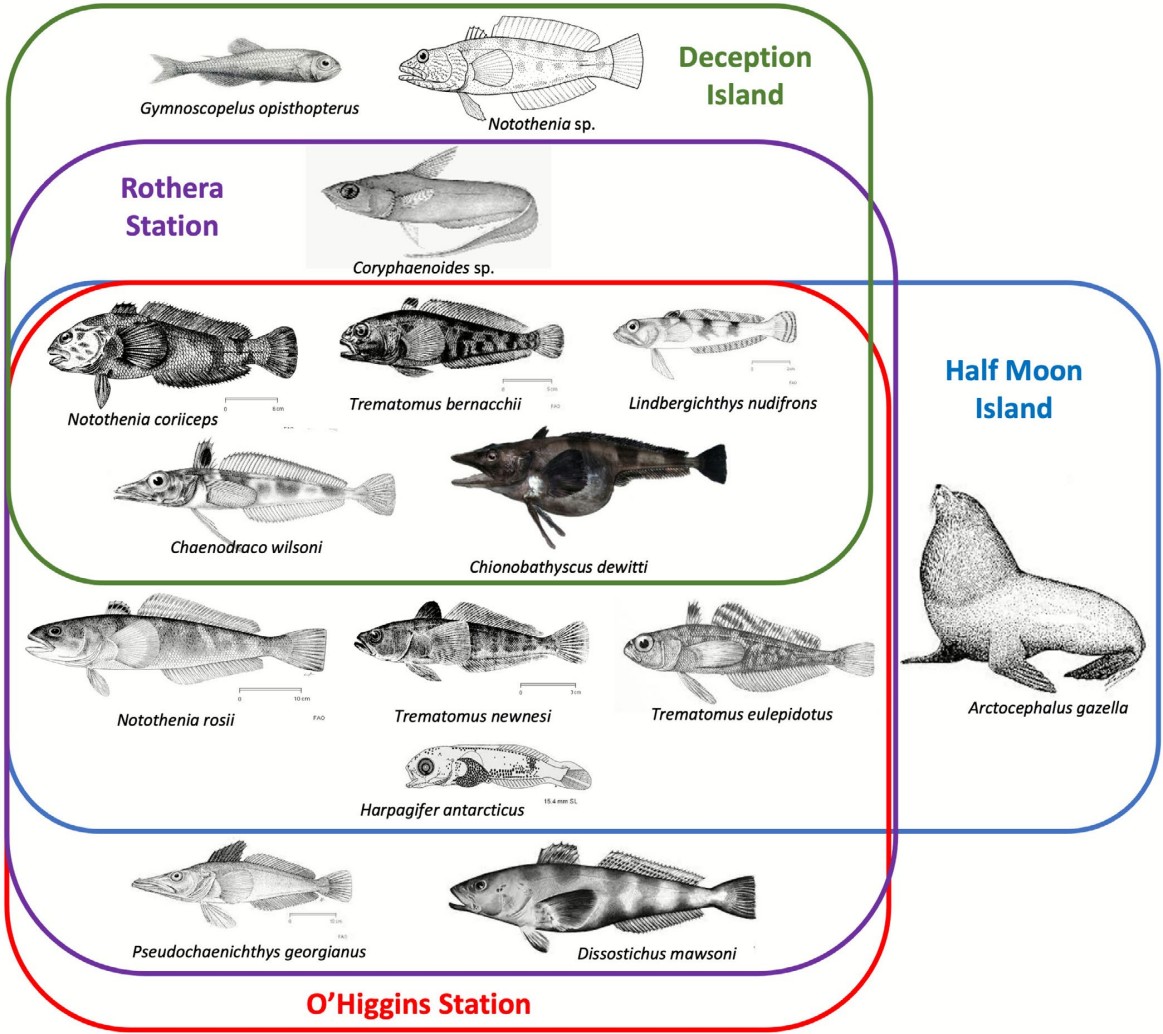
Fish community detected at each locality. All schemes were downloaded from FishBase.

Adelaide Island (Rothera Station) is the most southern and diverse place studied here. Although no differences were observed in the biodiversity indices within localities, when fish community structure was evaluated, slight differences were detected only with respect to O'Higgins. The main difference, apart from the contribution to biomass being almost triple in O'Higgins with respect to Rothera, was the absolute dominance of *N. coriiceps* in O'Higgins, followed by *N. rossi* and *T. newnesi* , while in Rothera, *L. nudifrons* was the most abundant species. It should be noted, also, that these two localities were the places with the highest differences in the number of detected reads between sponge and water samples. At the same time, Half Moon Island and O'Higgins displayed very similar fish communities, not endorsing the ichthyofaunistic zones, at least for nearshore shallow waters (Figure 7) (Kock 1992).

In inshore waters, the ecological role of demersal fish is arguably more important than that of krill (Barrera‐Oro 2002). Demersal fish are major consumers of benthic organisms and also feed on zooplankton (mainly krill in summer). They are links between lower and upper levels of the food web, and they are common prey of other fish, birds, and seals (Barrera‐Oro 2002). All the species detected in this study are endemic Antarctic species, widely distributed and known to occur in the studied region. Thus, this kind of investigation is particularly important in the light of increased human impact on the Antarctic marine ecosystem, currently being directly impacted by the commercial harvesting of fish and krill. Recognizing the strengths and limitations of eDNA metabarcoding as a non‐invasive and cost‐effective approach for monitoring marine fish communities will be useful for further ecosystem conservation strategies and biodiversity monitoring of the Southern Ocean.

## Author Contributions

**Carlos Angulo‐Preckler:** conceptualization (lead), data curation (lead), formal analysis (lead), investigation (lead), methodology (lead), visualization (lead), writing – original draft (lead). **Marta Turon:** data curation (supporting), formal analysis (supporting), methodology (supporting), visualization (supporting), writing – review and editing (equal). **Oriol Sacristan‐Soriano:** data curation (supporting), methodology (supporting), writing – review and editing (equal). **Kim Præbel:** funding acquisition (lead), resources (lead), supervision (supporting), writing – review and editing (equal). **Conxita Avila:** data curation (supporting), funding acquisition (supporting), project administration (lead), resources (supporting), supervision (supporting), writing – review and editing (equal). **Owen Simon Wangensteen:** data curation (supporting), formal analysis (supporting), methodology (lead), software (lead), supervision (lead), writing – review and editing (equal).

## Funding

This work was supported by Agència de Gestió d'Ajuts Universitaris i de Recerca (Grant 2021 SGR 01271), Universitetet i Tromsø.

## Conflicts of Interest

The authors declare no conflicts of interest.

## Supporting information


**Figure S1:** (a) Relationship between total MOTUs detection and number of total reads. Shadowed area represents 95% confidence interval. (b) Relationship between total reads (log transformed) and total detections per sample colored by sample type.
**Figure S2:** Bar plot showing the richness per sample. Y‐axis indicate the number of samples (replicates pooled) with the same number of MOTUs detected.
**Figure S3:** Total reads and biodiversity indices comparison between both pore sizes filters (5 and 0.22 μm). *Displayed significant differences with ANOVA test (*p* = 0.05).
**Figure S4:** Total reads per sample type (Water vs. Porifera) across different localities. Violin plots show the distribution of reads, overlaid boxplots indicate the median and interquartile range, and individual points represent each sample. Reads are plotted on a log10 scale to accommodate the wide range of sequencing depth. Statistical differences between Water and Porifera within each locality were assessed using Wilcoxon rank‐sum tests.
**Figure S5:** Technical replicate variability across sample types and localities. Violin plots show the distribution of coefficients of variation (CV) for technical replicates across four Antarctic localities. Jittered points represent individual technical replicate CVs. Wilcoxon rank‐sum tests compare CVs between water and sponge samples within each locality.
**Figure S6:** Total reads grouped by fish family. Percentages were displayed at the top of each bar.
**Figure S7:** Venn diagram representing the number of species identified at each locality and the overlap among them.
**Table S2:** Biodiversity indices by locality, by sample type, and by filter pore sizes. S; Species richness. H; Shannon biodiversity index. D; Simpson biodiversity index. Total reads.
**Table S3:** PERMANOVA test results. Factor; Sample type. Factor; Sponge species. Factor; Locality. All based in Bray–Curtis dissimilarity matrix.

**Table S1:** Summary of sequencing output per sample, including the number of raw reads obtained after demultiplexing, total reads retained after clustering, and final reads after contaminant removal.

## Data Availability

The data that support the findings of this study are available in the SRA repository of the NCBI at SRA bioproject, reference number PRJNA1017664. The ngsfilter tables containing the sample tags needed for demultiplexing the raw data and the sample metadata table are available from Mendeley Data: datasets/ww539fsxtj/1. The MJOLNIR pipeline (metabarcoding/MJOLNIR) was used for the bioinformatic analyses.
