# Supplementary material for: Are Sponges Good Natural Sentinels for Monitoring Fish Diversity in Antarctic Coastal Waters?
Source: Ecol Evol. 2025 Dec 16;15(12):e72684. doi: 10.1002/ece3.72684 (PMC12706525; doi:10.1002/ece3.72684)
Supplement: Supplementary file 1 — Figure S1: (a) Relationship between total MOTUs detection and number of total reads. Shadowed area represents 95% confidence interval. (b) Relationship between total reads (log transformed) and total detections per sample colored by sample type. Figure S2: Bar plot showing the richness per sample. Y‐axis indicate the number of samples (replicates pooled) with the same number of MOTUs detected. Figure S3: Total reads and biodiversity indices comparison between both pore sizes filters (5 and 0.22 μm). *Displayed significant differences with ANOVA test (p = 0.05). Figure S4: Total reads per sample type (Water vs. Porifera) across different localities. Violin plots show the distribution of reads, overlaid boxplots indicate the median and interquartile range, and individual points represent each sample. Reads are plotted on a log10 scale to accommodate the wide range of sequencing depth. Statistical differences between Water and Porifera within each locality were assessed using Wilcoxon rank‐sum tests. Figure S5: Technical replicate variability across sample types and localities. Violin plots show the distribution of coefficients of variation (CV) for technical replicates across four Antarctic localities. Jittered points represent individual technical replicate CVs. Wilcoxon rank‐sum tests compare CVs between water and sponge samples within each locality. Figure S6: Total reads grouped by fish family. Percentages were displayed at the top of each bar. Figure S7: Venn diagram representing the number of species identified at each locality and the overlap among them. Table S2: Biodiversity indices by locality, by sample type, and by filter pore sizes. S; Species richness. H; Shannon biodiversity index. D; Simpson biodiversity index. Total reads. Table S3: PERMANOVA test results. Factor; Sample type. Factor; Sponge species. Factor; Locality. All based in Bray–Curtis dissimilarity matrix. [file ECE3-15-e72684-s001.docx]

**Supplementary material**

**Figure S1. a)** Relationship between total MOTUs detection and number of total reads. Shadowed area represents 95% confidence interval. **b)** Relationship between total reads (log transformed) and total detections per sample colored by sample type.

**Figure S2.** Bar plot showing the richness per sample. Y-axis indicate the number of samples (replicates pooled) with the same number of MOTUs detected.

**Figure S3.** Total reads and biodiversity indices comparison between both pore sizes filters (5 and 0.22 µm). * Displayed significant differences with ANOVA test (*p* = 0.05).

**Figure S4.** Total reads per sample type (Water vs. Porifera) across different localities. Violin plots show the distribution of reads, overlaid boxplots indicate the median and interquartile range, and individual points represent each sample. Reads are plotted on a log10 scale to accommodate the wide range of sequencing depth. Statistical differences between Water and Porifera within each locality were assessed using Wilcoxon rank-sum tests.

**Figure S5.** Technical replicate variability across sample types and localities. Violin plots show the distribution of coefficients of variation (CV) for technical replicates across four Antarctic localities. Jittered points represent individual technical replicate CVs. Wilcoxon rank-sum tests compare CVs between water and sponge samples within each locality.

**Figure S6.** Total reads grouped by fish family. Percentages were displayed at the top of each bar.

**Figure S7.** Venn diagram representing the number of species identified at each locality and the overlap among them.

**Table S1.** Summary of sequencing output per sample, including the number of raw reads obtained after demultiplexing, total reads retained after clustering, and final reads after contaminant removal.

**Table S2.** Biodiversity indices by locality, by sample type, and by filter pore sizes. S; Species richness. H; Shannon biodiversity index. D; Simpson biodiversity index. Total reads.

**Table S3.** PERMANOVA test results. Factor; Sample type. Factor; Sponge species. Factor; Locality. All based in Bray-Curtis dissimilarity matrix.

**Figure S1.**


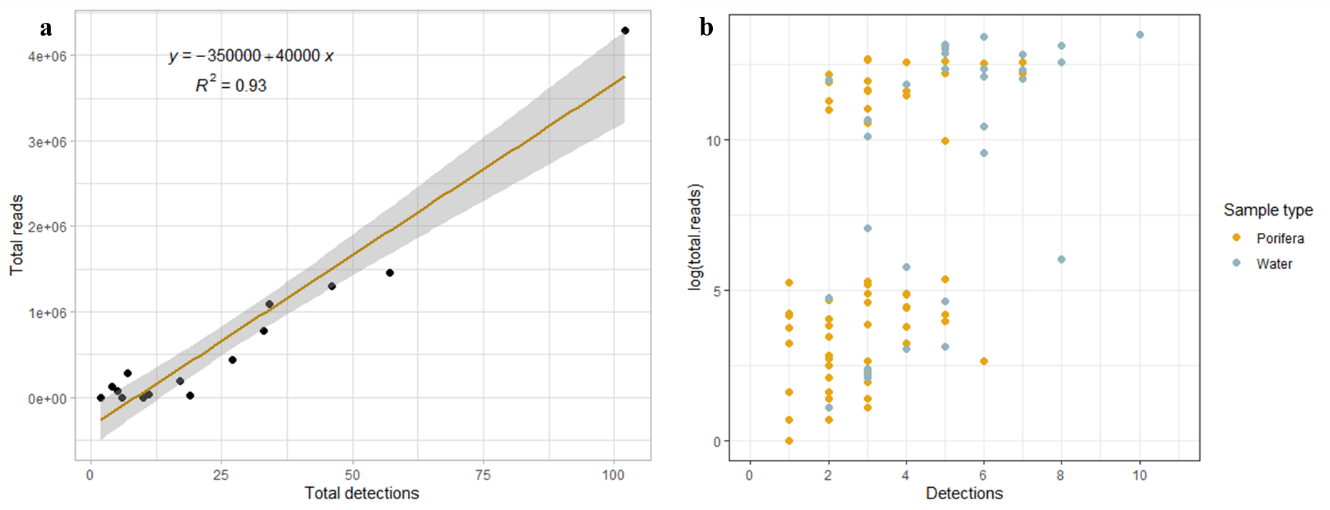


**Figure S2.**

**
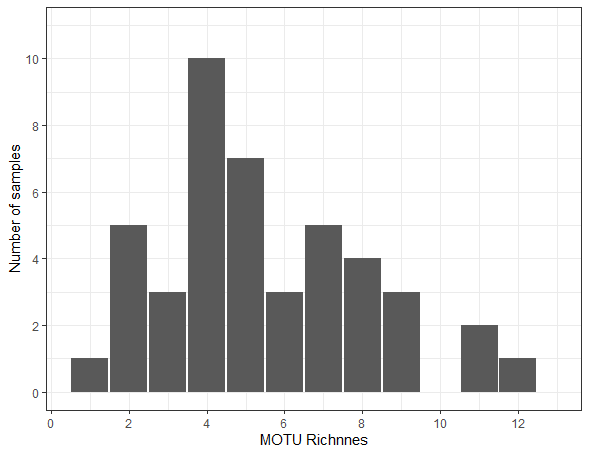
**

**Figure S3.**

**Figure S4**

**
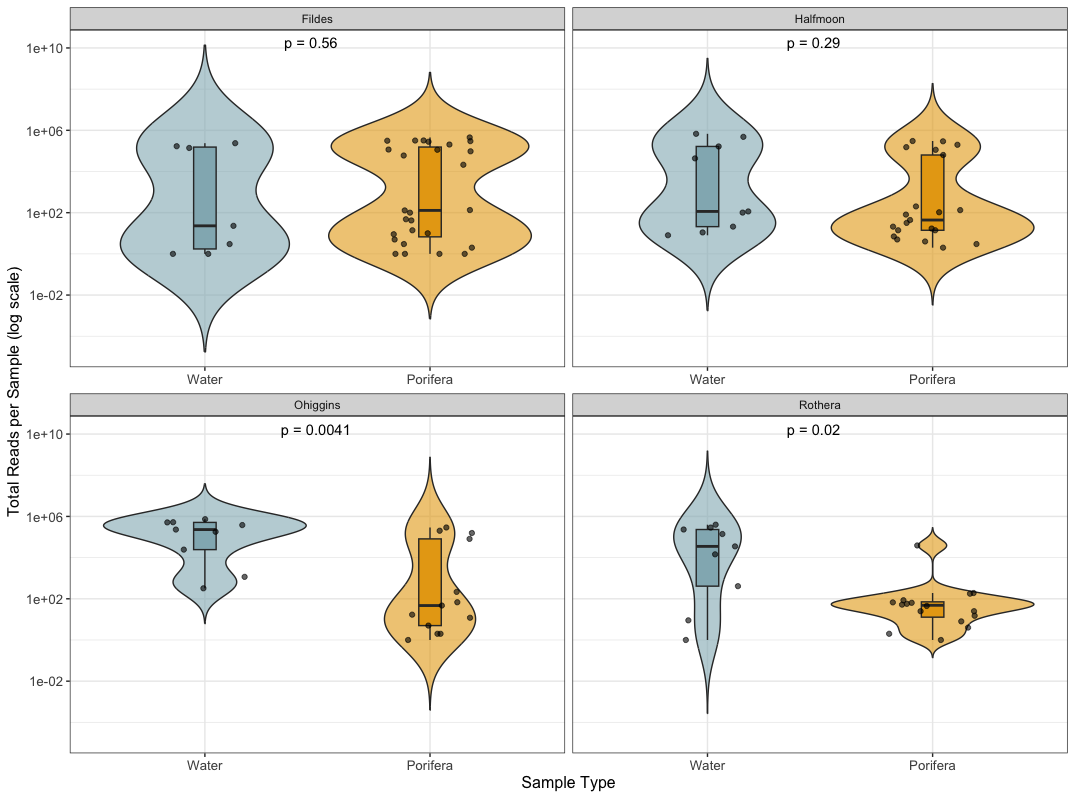
**

**Figure S5**

**
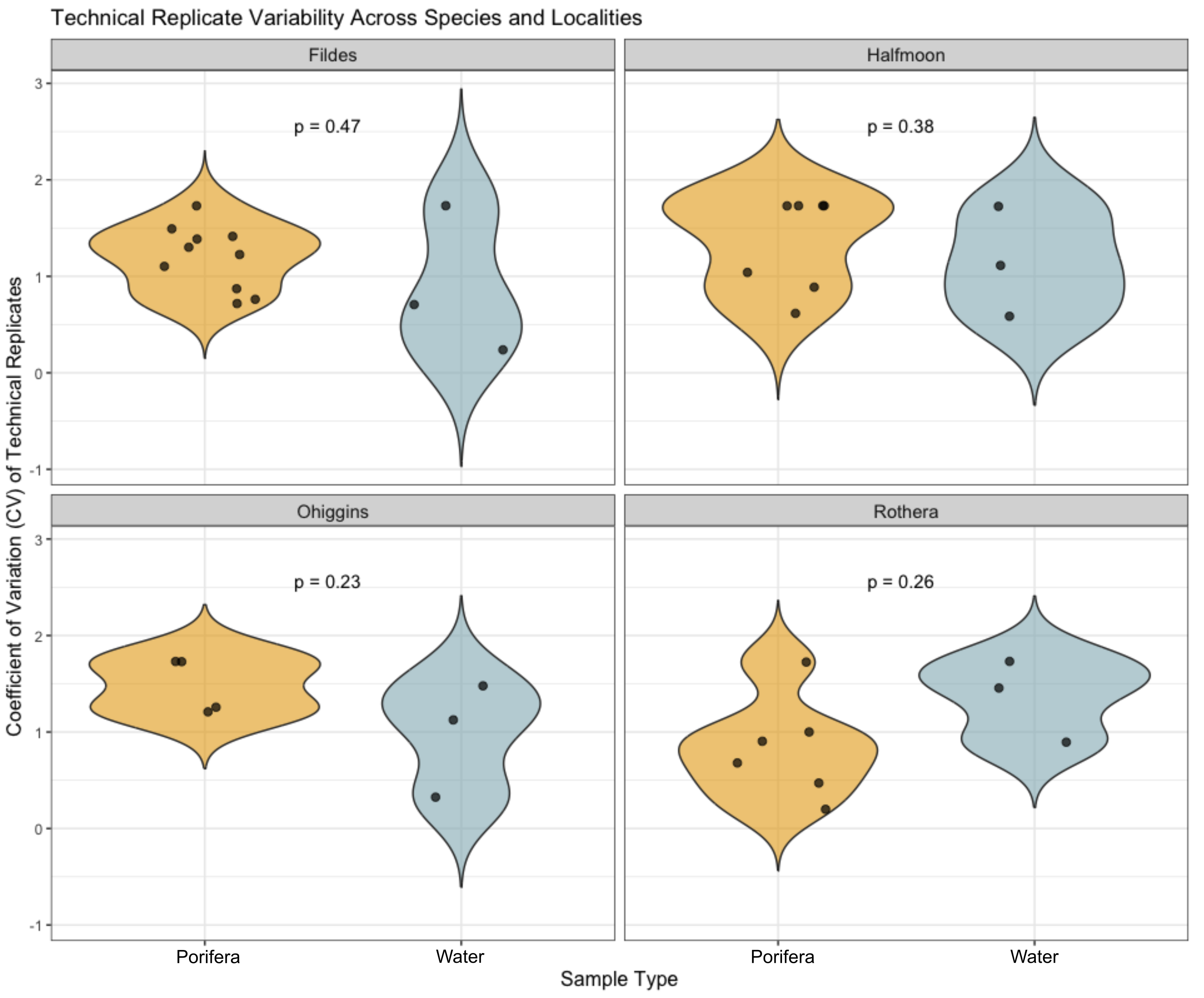
**

**Figure S6.**

**
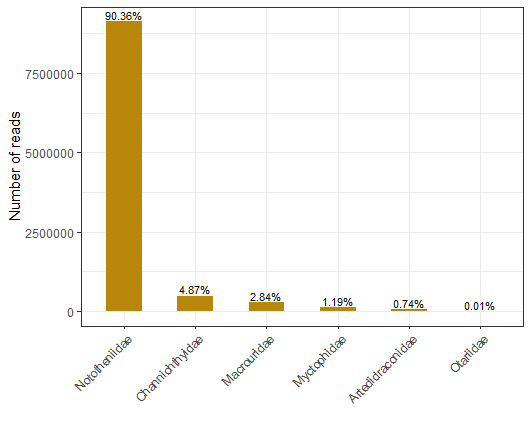
**

**Figure S7.**

**
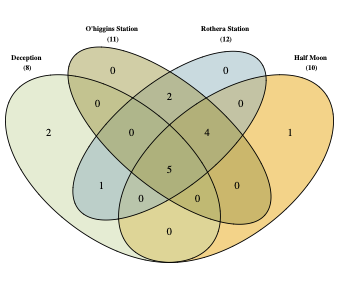
**

**Table S2**

| **Localities** | **S** | **H** | **D** | **Total reads** |
| --- | --- | --- | --- | --- |
| Deception | 3.91 ± 1.34 | 0.513 ± 0.401 | 0.294 ± 0.236 | 92236 ± 131201 |
| Halfmoon | 3.54 ± 1.47 | 0.567 ± 0.407 | 0.320 ± 0.222 | 82932 ± 162683 |
| O’Higgins | 4.56 ± 2.48 | 0.546 ± 0.321 | 0.325 ± 0.190 | 150733 ± 213990 |
| Rothera | 4 ± 2.21 | 0.593 ± 0.421 | 0.337 ± 0.239 | 45840 ± 104877 |
| **Sample type** |  |  |  |  |
| Porifera | 3.31 ± 1.46 | 0.520 ± 0.399 | 0.293 ± 0.242 | 58223 ± 109194 |
| Water | 5.25 ± 1.97 | 0.621 ± 0.355 | 0.341 ± 0.219 | 164793 ± 214519 |
| **Filters (µm)** |  |  |  |  |
| 5 | 4 ± 1.73 | 0.508 ± 0.343 | 0.295 ± 0.206 | 146438 ± 206603 |
| 0.22 | 3.04 ± 1.73 | 0.548 ± 0.365 | 0.330 ± 0.220 | 52164 ± 94704 |

**Table S3**

| Factor | Df | Sums Of Sqs | Mean Sqs | F.Model | R2 | Pr(>F) |
| --- | --- | --- | --- | --- | --- | --- |
| *Sample.type* | 1 | 0.939 | 0.93887 | 2.3285 | 0.02794 | 0.0189 * |
| *Residuals* | 81 | 32.659 | 0.40320 |  | 0.97206 |  |
| *Total* | 82 | 33.598 |  |  | 1.0000 |  |
|  |  |  |  |  |  |  |
| Factor | Df | Sums Of Sqs | Mean Sqs | F.Model | R2 | Pr(>F) |
| *Species* | 4 | 1.9503 | 0.48756 | 1.2549 | 0.22796 | 0.1588 |
| *Residuals* | 17 | 6.6049 | 0.38852 |  | 0.77204 |  |
| *Total* | 21 | 8.5551 |  |  | 1.0000 |  |
|  |  |  |  |  |  |  |
| Factor | Df | Sums Of Sqs | Mean Sqs | F.Model | R2 | Pr(>F) |
| *Locality* | 3 | 2.010 | 0.6699 | 1.6925 | 0.06854 | 0.0109 * |
| *Residuals* | 69 | 27.315 | 0.3958 |  | 0.93146 |  |
| *Total* | 72 | 29.325 |  |  | 1.0000 |  |

PERMANOVA analysis for Bray-Curtis distance using relative abundance.
